# Multi-analyte profile analysis of plasma immune proteins: altered expression of peripheral immune factors is associated with neuropsychiatric symptom severity in adults with and without chronic hepatitis C virus infection

**DOI:** 10.1002/brb3.200

**Published:** 2013-12-29

**Authors:** Marilyn Huckans, Bret E Fuller, Hannah Olavarria, Anna W Sasaki, Michael Chang, Kenneth D Flora, Michael Kolessar, Daniel Kriz, Jeanne R Anderson, Arthur A Vandenbark, Jennifer M Loftis

**Affiliations:** 1 Research & Development Service, Portland VA Medical Center3710 SW U.S. Veterans Hospital Rd., Portland, Oregon, 97239, USA; 2 Mental Health and Clinical Neurosciences Division, Portland VA Medical Center3710 SW U.S. Veterans Hospital Rd., Portland, Oregon, 97239, USA; 3 Department of Psychiatry, Oregon Health & Science University3181 SW Sam Jackson Park Rd., Portland, Oregon, 97239, USA; 4 Gastroenterology Service, Portland VA Medical Center3710 SW US Veterans Hospital Rd., Portland, Oregon, 97239, USA; 5 Department of Internal Medicine, Oregon Health & Science University3181 SW Sam Jackson Park Rd., Portland, Oregon, 97239, USA; 6 Portland Gastroenterology Division, Oregon Clinic9280 SE Sunnybrook Blvd., Clackamas, Oregon, 97015, USA; 7 School of Professional Psychology, Pacific University190 SE 8th Ave., Hillsboro, Oregon, 97123, USA; 8 Department of Neurology, Oregon Health & Science University3181 SW Sam Jackson Park Rd., Portland, Oregon, 97239, USA; 9 Department of Molecular Microbiology and Immunology, Oregon Health & Science University3181 SW Sam Jackson Park Rd., Portland, Oregon, 97239, USA

**Keywords:** Anxiety, biological markers, chronic infection, cytokines, depression, fatigue, pain

## Abstract

**Background:**

The purpose of this study was to characterize hepatitis C virus (HCV)-associated differences in the expression of 47 inflammatory factors and to evaluate the potential role of peripheral immune activation in HCV-associated neuropsychiatric symptoms—depression, anxiety, fatigue, and pain. An additional objective was to evaluate the role of immune factor dysregulation in the expression of specific neuropsychiatric symptoms to identify biomarkers that may be relevant to the treatment of these neuropsychiatric symptoms in adults with or without HCV.

**Methods:**

Blood samples and neuropsychiatric symptom severity scales were collected from HCV-infected adults (HCV+, *n* = 39) and demographically similar noninfected controls (HCV−, *n* = 40). Multi-analyte profile analysis was used to evaluate plasma biomarkers.

**Results:**

Compared with HCV− controls, HCV+ adults reported significantly (*P* < 0.050) greater depression, anxiety, fatigue, and pain, and they were more likely to present with an increased inflammatory profile as indicated by significantly higher plasma levels of 40% (19/47) of the factors assessed (21%, after correcting for multiple comparisons). Within the HCV+ group, but not within the HCV− group, an increased inflammatory profile (indicated by the number of immune factors > the LDC) significantly correlated with depression, anxiety, and pain. Within the total sample, neuropsychiatric symptom severity was significantly predicted by protein signatures consisting of 4–10 plasma immune factors; protein signatures significantly accounted for 19–40% of the variance in depression, anxiety, fatigue, and pain.

**Conclusions:**

Overall, the results demonstrate that altered expression of a network of plasma immune factors contributes to neuropsychiatric symptom severity. These findings offer new biomarkers to potentially facilitate pharmacotherapeutic development and to increase our understanding of the molecular pathways associated with neuropsychiatric symptoms in adults with or without HCV.

## Introduction

Approximately 2–3% of adults worldwide are chronically infected with the hepatitis C virus (HCV; Lavanchy 2009). Although the majority of adults with HCV avoid these serious hepatic complications and live a full life span, a growing body of literature demonstrates that, even in the absence of antiviral treatment for HCV—which is well known to cause depression and other neuropsychiatric symptoms (e.g., Loftis and Hauser 2004; Udina et al. 2012)—many of these individuals suffer from a range of extrahepatic manifestations including chronic neuropsychiatric impairments such as depression, anxiety, fatigue, pain, and cognitive deficits. For example, in one study (*n* = 8224), 67% of adults with HCV were found to have comorbid chronic pain diagnoses documented in their medical record (Whitehead et al. 2008). Another study (*n* = 1614) found that 53% reported general fatigue and 17% reported severe fatigue that was debilitating (Poynard et al. 2002). In a prospective study of 293 adults with HCV, 95% were found to have a current or past history of at least one psychiatric disorder; the most common of these conditions was depression, with 81% reporting a history of depression, and 35% reporting current depression rating scale scores in the moderate to severe range (Fireman et al. 2005). Depressive symptoms in particular are important contributors to functional disability and decreased health-related quality of life in patients with HCV (Dwight et al. 2000; Rowan et al. 2005; Dan et al. 2006), and moderate to severe depressive symptoms are also a common reason for postponing or excluding patients from antiviral therapy (Rowan et al. 2005). Although anxiety disorders are not as well studied in this population, Golden et al. (2005; *n* = 90) found that 24% of individuals who were about to initiate antiviral treatment for HCV met criteria for an anxiety disorder within the previous month, 86% of whom were previously undiagnosed. Another study (*n* = 176) found that 10% of those about to initiate antiviral therapy for HCV met criteria for a lifetime history of an anxiety disorder (Martin-Santos et al. 2008). Collectively, these findings suggest that HCV is associated with a constellation or syndrome of neuropsychiatric impairments which may, therefore, stem from a common etiology (e.g., chronic immune activation on brain function). Indeed, immune activation, rather than an epiphenomenon, is now considered to be a causal risk factor for the development of depression in some individuals (Wichers et al. 2006).

Although transient activation of the immune system and related sickness behaviors (e.g., decreased motility, increased fatigue and sleep, reduced appetite, increased sensitivity to pain, decreased motivation or interest, decreased sexual activity, hyperthermia; Dantzer and Kelley 2007) may be adaptive in the context of acute infection, it is thought that chronic dysregulation of these immune factors, such as in the context of cytokine treatments for HCV or cancer (i.e., interferon-based therapies), may contribute to the development of long-term neuropsychiatric disorders and symptoms (McAfoose and Baune 2009; Loftis et al. 2010; Capuron and Miller 2011). Similarly, elevations of proinflammatory cytokines (e.g., interleukin [IL]-1, IL-6, tumor necrosis factor [TNF]) and chemokines (e.g., RANTES [regulated upon activation, normal T-cell expressed, and secreted]) are evidenced in patients diagnosed with a range of chronic neuropsychiatric disorders including depression (Maes et al. 1995; Levine et al. 1999; Owen et al. 2001; Hestad et al. 2003; Loftis et al. 2008; Howren et al. 2009; Leonard and Maes 2012), anxiety (Hoge et al. 2009; Hou and Baldwin 2012), chronic fatigue syndrome (Arnett and Clark 2012), cancer-related fatigue and cognitive impairment (Meyers et al. 2005), pain disorders (Slade et al. 2011; Alexander et al. 2012), and age-related cognitive decline and dementia (Yaffe et al. 2004; Britschgi and Wyss-Coray 2009; Marksteiner et al. 2011; Corona et al. 2012). Collectively, these studies highlight the impact that immune activation and immune factor dysregulation (both peripherally and centrally) can have on central nervous system (CNS) function.

Emerging evidence suggests that the HCV itself may directly contribute to increased immune activation and proinflammatory cytokine expression in the CNS. Hepatitis C viral sequences and proteins have been found in brain macrophage/microglia cells, and activation of these brain cells in HCV+ patients is associated with higher expression of messenger ribonucleic acid (mRNA) transcripts for key immune activation cytokines (e.g., IL-1 and TNF-*α*) than in HCV− control patients (Wilkinson et al. 2010). When analyzing a small panel of one or several blood immune factors, previous studies have revealed significantly increased levels of specific blood immune factor levels, including IL-6, IL-18, IL-10, IL-4, TNF-*α*, and RANTES, in untreated HCV+ adults compared with uninfected (HCV−) controls (Abayli et al. 2003; Vecchiet et al. 2005; Falasca et al. 2006; Grungreiff et al. 2009; Tawadrous et al. 2012). Moreover, in two small studies, peripheral immune factor levels were shown to be significantly associated with neuropsychiatric impairments in untreated HCV+ adults. Hilsabeck et al. (2010) examined serum levels of IFN-*α*, IL-6, and TNF-*α* in relation to cognition; in HCV+ adults with detectable IFN-*α* levels (*n* = 17), higher IL-6 levels correlated with worse visual memory and sustained visual attention, and higher TNF-*α* levels correlated with worse visual memory and visual perception. Loftis et al. (2008) examined plasma levels of IL-1*β*, TNF-*α*, and IL-10 in relation to depression and found that, in untreated HCV+ adults (*n* = 16), elevations in IL-1*β* and TNF-*α* correlated with more severe depressive symptoms. Both studies, however, were limited by small sample sizes and investigated only a few immune factors. It was recently reported that studies like these “highlight the need to develop a biomarker panel for depression that aims to profile diverse peripheral factors that together provide a biological signature of MDD (major depressive disorder) subtypes as well as treatment response” (Schmidt et al. 2011). Therefore, replication is required with a larger array of immune factors. Because the expression levels of cytokines and chemokines (inflammatory markers) are heterogeneous, it is not likely that a single cytokine or inflammatory marker will differentiate between individuals with or without depressive symptoms, for example. Rather, the person's composite “profile” or protein “signature” may serve to successfully identify biomarkers of depression and other neuropsychiatric impairments.

The primary objective of this study was to characterize HCV-associated differences in the expression of a large array of peripheral immune proteins using multi-analyte profile (MAP) analysis of 47 plasma immune factors (see Table 1 for a list of factors), and to evaluate the potential role of peripheral immune activation in HCV-associated neuropsychiatric impairments—depression, anxiety, fatigue, and pain. Because of the high rates of comorbid psychiatric disorders among individuals with HCV (Nelligan et al. 2008), the neuropsychiatric effects of HCV are of particular concern. Given that cytokines and chemokines can influence neurotransmitter systems and contribute to behavioral changes, increasingly, immune factors are also thought to play a role in the development of neuropsychiatric symptoms—even in individuals without preexisting immune compromise (e.g., Maes et al. 2011; Salim et al. 2012; Anderson et al. 2013). Thus, an additional objective was to use MAP analysis to evaluate the effects of immune factor dysregulation on neuropsychiatric function in order to identify novel biomarkers that might be relevant to the discovery and development of new treatments for neuropsychiatric symptoms in adults with or without HCV. To our knowledge, this study is among the first to apply MAP analysis of a large array of immune factors to evaluate the association between altered plasma immune factor expression and the severity of depression, anxiety, fatigue, and pain symptoms.

**Table 1:** Between-group comparisons of immune factor profiles in adults with hepatitis C virus (HCV+, *n* = 39) and without (HCV−, *n* = 40)

Factor (abbreviation)	Units	LDC^1^	HCV+ % ≥ LDC^2^	HCV− % ≥ LDC^2^	*z*-value^3^	*P*-value^3^	HCV+ median (IQR)^4^	HCV− median (IQR)^4^	*P*-value^4^
Alpha-2-macroglobulin (A2Macro)	mg/mL	0.0204	100	100	0.00	>0.999	0.633 (0.169)	0.514 (0.181)	<0.001^***^
Alpha-1-antitrypsin (AAT)	mg/mL	0.0029	100	100	0.00	>0.999	1.020 (0.250)	0.891 (0.235)	0.002^**^
Beta-2-microglobulin (B2M)	μg/mL	0.0581	100	100	0.00	>0.999	0.928 (0.294)	0.653 (0.236)	<0.001^***^
Brain-derived neurotrophic factor (BDNF)	ng/mL	0.0197	100	100	0.00	>0.999	5.070 (2.790)	5.290 (2.170)	0.164
Complement C3 (C3)	mg/mL	0.0035	100	100	0.00	>0.999	0.373 (0.139)	0.395 (0.098)	0.239
C-reactive protein (CRP)	μg/mL	0.0231	100	100	0.00	>0.999	0.536 (0.871)	1.310 (2.009)	0.004^**^
Eotaxin-1	pg/mL	100.0000	92	83	1.31	0.189	197.000 (95.000)	167.000 (88.300)	0.317
Factor VII	ng/mL	2.8000	100	100	0.00	>0.999	687.000 (231.000)	564.500 (217.000)	0.017^*^
Fibrinogen	mg/mL	0.0176	100	100	0.00	>0.999	2.980 (0.720)	2.950 (0.940)	0.673
Ferritin (FRTN)	ng/mL	2.4900	100	100	0.00	>0.999	73.100 (110.500)	59.200 (72.430)	0.027^**^
*Granulocyte-macrophage colony-stimulating factor (GM-CSF)*	*pg/mL*	*8.9300*	*0*	*0*	*0.00*	*>0.999*	*<0.001 (<0.001)*	*<0.001 (<0.001)*	0.039^*^
Haptoglobin	mg/mL	0.0071	95	100	1.43	0.152	0.478 (0.499)	0.325 (0.462)	0.344
Intercellular adhesion molecule 1 (ICAM-1)	ng/mL	2.3900	100	98	1.00	0.317	160.000 (96.000)	81.750 (25.700)	<0.001^***^
*Interferon gamma (IFN-gamma)*	*pg/mL*	*1.8800*	*0*	*0*	*0.00*	*>0.999*	*<0.001 (<0.001)*	*<0.001 (<0.001)*	*0.355*
*Interleukin-1 alpha (IL-1α)*	*ng/mL*	*0.0073*	*0*	*0*	*0.00*	*>0.999*	*<0.001 (<0.001)*	*<0.001 (0.001)*	*0.491*
*Interleukin-1 beta (IL-1β)*	*pg/mL*	*1.7900*	*0*	*0*	*0.00*	*>0.999*	*<0.001 (<0.001)*	*<0.001 (<0.001)*	*>0.999*
Interleukin-10 (IL-10)	pg/mL	1.3900	74	43	3.00	0.003^**^	2.530 (2.240)	1.090 (1.900)	0.007^**^
*Interleukin-12 subunit p40 (IL-12p40)*	*ng/mL*	*0.1120*	*0*	*0*	*0.00*	*>0.999*	*<0.001 (<0.001)*	*<0.001 (<0.001)*	*>0.999*
*Interleukin-12 subunit p70 (IL-12p70)*	*pg/mL*	*23.1000*	*0*	*0*	*0.00*	*>0.999*	*<0.001 (<0.001)*	*<0.001 (<0.001)*	*0.806*
Interleukin-15 (IL-15)	ng/mL	0.4400	72	60	1.10	0.271	0.782 (0.571)	0.506 (0.782)	0.118
*Interleukin-17 (IL-17)*	*pg/mL*	*6.5800*	*0*	*0*	*0.00*	*>0.999*	*1.980 (2.050)*	*1.980 (1.340)*	*0.823*
Interleukin-18 (IL-18)	pg/mL	12.9000	100	100	0.00	>0.999	176.000 (101.000)	111.50 (54.700)	<0.001^***^
*Interleukin-1 receptor antagonist (IL-1ra)*	*pg/mL*	*125.0000*	*0*	*3*	*1.00*	*0.317*	*<0.001 (54.900)*	*<0.001 (43.000)*	*0.597*
*Interleukin-2 (IL-2)*	*pg/mL*	*7.9000*	*0*	*0*	*0.00*	*>0.999*	*<0.001 (<0.001)*	*<0.001 (<0.001)*	*0.373*
Interleukin-23 (IL-23)	ng/mL	0.8180	21	15	0.63	0.526	<0.001 (0.647)	<0.001 (0.623)	0.128
*Interleukin-3 (IL-3)*	*ng/mL*	*0.0201*	*3*	*0*	*1.50*	*0.133*	*<0.001 (<0.001)*	*<0.001 (<0.001)*	0.022^*^
*Interleukin-4 (IL-4)*	*pg/mL*	*18.0000*	*0*	*0*	*0.00*	*>0.999*	*<0.001 (<0.001)*	*<0.001 (<0.001)*	*0.584*
Interleukin-5 (IL-5)	pg/mL	3.4700	5	5	0.03	0.979	<0.001 (<0.001)	<0.001 (<0.001)	0.979
*Interleukin-6 (IL-6)*	*pg/mL*	*4.1150*	*0*	*0*	*0.00*	*>0.999*	*1.250 (1.670)*	*1.040 (1.425)*	0.026^*^
Interleukin-7 (IL-7)	pg/mL	7.6400	15	8	1.09	0.275	<0.001 (4.160)	<0.001 (4.160)	0.588
Interleukin-8 (IL-8)	pg/mL	1.8800	100	100	0.00	>0.999	8.470 (5.600)	5.520 (2.440)	<0.001^***^
Monocyte chemotactic protein 1 (MCP-1)	pg/mL	4.7800	100	100	0.00	>0.999	112.000 (47.800)	98.100 (53.700)	0.224
Macrophage inflammatory protein-1 alpha (MIP-1*α*)	pg/mL	22.9000	44	13	3.23	0.001^***^	21.200 (16.000)	9.630 (17.380)	<0.001^***^
Macrophage inflammatory protein-1 beta (MIP-1*β*)	pg/mL	12.2000	100	100	0.00	>0.999	142.000 (76.000)	103.500 (56.000)	0.006^**^
*Matrix metalloproteinase-2 (MMP-2)*	*ng/mL*	*6.0100*	*3*	*0*	*1.00*	*0.318*	*1.540 (2.420)*	*0.575 (1.540)*	*0.085*
Matrix metalloproteinase-3 (MMP-3)	ng/mL	0.0507	100	100	0.00	>0.999	2.840 (1.850)	2.985 (2.057)	0.914
Matrix metalloproteinase-9 (MMP-9)	ng/mL	20.7000	10	13	0.31	0.757	9.350 (16.100)	3.405 (16.100)	0.353
T-Cell-specific protein regulated on activation, normal T-cell expressed and secreted (RANTES)	ng/mL	0.0592	100	100	0.00	>0.999	9.200 (6.220)	10.400 (4.295)	0.135
Stem cell factor (SCF)	pg/mL	97.8000	90	80	1.21	0.228	144.000 (72.000)	125.000 (37.000)	0.069
Tissue inhibitor of metalloproteinases 1 (TIMP-1)	ng/mL	3.8300	100	100	0.00	>0.999	56.200 (27.400)	45.450 (8.800)	<0.001^***^
Tumor necrosis factor alpha (TNF-*α*)	pg/mL	1.0300	82	55	2.68	0.008^**^	1.690 (1.040)	1.210 (1.910)	0.003^**^
*Tumor necrosis factor beta (TNF-β)*	*pg/mL*	*61.3000*	*3*	*0*	*1.00*	*0.318*	*<0.001 (<0.001)*	*0.336 (<0.001)*	*0.719*
Tumor necrosis factor receptor 2 (TNFR2)	ng/mL	0.3680	100	100	0.00	>0.999	3.770 (1.480)	2.480 (0.880)	<0.001^***^
Vascular cell adhesion molecule-1 (VCAM-1)	ng/mL	1.7060	100	100	0.00	>0.999	417.000 (180.000)	283.500 (104.000)	<0.001^***^
Vitamin D-binding protein (VDBP)	μg/mL	5.6200	100	100	0.00	>0.999	165.000 (164.300)	170.000 (116.300)	0.953
Vascular endothelial growth factor (VEGF)	pg/mL	9.0700	100	100	0.00	>0.999	176.000 (61.000)	159.500 (53.000)	0.103
von Willebrand Factor (vWF)	μg/mL	0.1410	100	100	0.00	>0.999	29.800 (15.600)	21.700 (13.200)	<0.001^***^

1 The LDC is defined as the mean ± 3 standard deviations (SDs) of 20 blank readings as provided by Myriad Rules Based Medicine, Inc.

2 Percentage of adults within each group with levels ≥ LDC are reported. For regression modeling, analytes detected in 5% or fewer of samples were excluded from analyses. These excluded analytes are shown in italics.

3 The percentages of immune factors ≥ LDC were compared across groups with tests of two proportions, and the *z* and *P*-values are reported.

4 Between-group comparisons of plasma immune factor levels were computed with Mann–Whitney *U*-tests, and the medians and interquartile ranges (IQRs) within each group are reported.

^*^P ≤ 0.050; ^**^P ≤ 0.010; ^***^P ≤ 0.001. Shading denotes immune factors that remained significantly different between groups following a Bonferroni correction for multiple comparisons, ^***^P ≤ 0.001.

## Method

### Participants

A total of 79 adults were recruited from the Portland, Oregon area and assigned to one of two groups: (1) adults with chronic HCV (HCV+, *n* = 39), as indicated by a detectable HCV viral load based on polymerase chain reaction (PCR) tests, and (2) adults with no reported history of HCV and a currently negative HCV antibody test (HCV−, *n* = 40). Participants in the HCV+ group were recruited from one of several area hepatology clinics through referral by the hepatologist, study advertisements posted in the hepatology clinic, announcements at the clinics' HCV education classes, or mailings to patients who had previously participated in HCV research. HCV− controls were recruited via study advertisements posted in the hospitals that housed the hepatology clinics, through word of mouth via providers in those facilities, or through study advertisements posted in local newspapers and websites. Participants were excluded if they met any of the following criteria: (1) History of antiviral therapy or chemotherapy for any purpose. (2) History of a major medical condition, or currently unstable medical condition, that was likely to be associated with severe neurological, cognitive, or immune dysfunction at the time of enrollment (e.g., stroke, seizures, brain tumors, Parkinson's disease, neurodegenerative dementia, mental retardation, hepatic encephalopathy, human immunodeficiency virus [HIV]). In the interest of generalizability to typical HCV+ populations, participants with common well-controlled or stable conditions were included as long as severe cognitive or immunological effects were not suspected at the time of enrollment (e.g., well-controlled diabetes, hypertension, or asthma). (3) History of traumatic brain injury with known loss of consciousness ≥30 min. (4) Use of alcohol, illicit substances, or medications with acute cognitive effects such as sedation or intoxication (e.g., benzodiazepines, opiates, muscle relaxants, psychostimulants) on the day of testing, or chronic use of medications associated with long-term cognitive or immune effects (e.g., topiramate, remicade, anticholinergics, steroids). (5) Decompensated liver cirrhosis, clinically determined by a hepatologist (Anna W. Sasaki) based on clinical indicators, medical record, biopsy results (if available), and a battery of standard medical laboratory tests (liver panel, complete blood count [CBC], International Normalized Ratio [INR], ammonia). (6) Current pregnancy. (7) History of schizophrenia or schizoaffective disorder, OR, current psychotic or manic episode, OR currently unstable and severe psychiatric disorder. In the interest of generalizability to typical HCV+ populations, patients with mild but stable depression, anxiety, or posttraumatic stress disorder (PTSD) were included as long as their symptoms did not preclude valid participation. (8) Alcohol or drug dependence within the past year (except nicotine or caffeine), based on Diagnostic and Statistical Manual of Mental Disorders, Fourth Edition (DSM-IV) criteria (American Psychiatric Association 2000), confirmed with the Mini-International Neuropsychiatric Interview (MINI; Sheehan et al. 1998).

### Procedures

All research was conducted with permission from the Portland Veterans Affairs Medical Center (PVAMC)'s Institutional Review Board and in accordance with the Helsinki Declaration as revised 1989. All patients were paid $75 to complete the following study procedures: clinical interview, comprehensive medical record review, a battery of questionnaires to assess severity of depression, anxiety, fatigue, and pain, and blood sample collection for standard medical laboratory tests (liver panel, CBC, INR, ammonia, HIV antibody screening, HCV testing [HCV antibody, followed by HCV recombinant immunoblot assay, HCV PCR Qualitative, and HCV PCR Quantitative if HCV antibody positive]) and multiplex assessment. Blood samples were collected by certified phlebotomists in the PVAMC medical laboratory. All other study procedures were administered by one of four study personnel (H. Olavarria, D. Kriz, M. Kolessar, J. R. Anderson) who were trained and supervised by a clinical neuropsychologist (M. Huckans). To ensure accuracy, all neuropsychiatric measures were scored and then re-scored by separate study personnel. All study data were entered into a database initially and then double-checked by separate study personnel prior to analyses.

Clinical interviews were conducted using a structured case report form, developed specifically for this study, including prompts to screen patients based on each inclusion criteria, gather relevant demographic data, assess for a full range of current and past Axis I psychiatric and substance use disorders using DSM-IV (American Psychiatric Association 2000) criteria and the MINI (Sheehan et al. 1998), evaluate for history of head injuries, and record a comprehensive list of current and previous medical conditions and medications. Study personnel additionally reviewed each participant's complete electronic medical record if treated at PVAMC, or the medical records forwarded by a treating hepatologist or primary care provider if treated elsewhere to cross validate the psychiatric, substance use, and medical history gathered in the clinical interview.

### Questionnaires

#### Depression

Beck Depression Inventory, Second Edition (BDI-II; Beck 1996). A well-validated 21-item measure of depression severity. As previously described (Patterson et al. 2011), we conducted a factor analysis of BDI-II data from a large sample of 671 HCV+ patients which yielded a two-factor model and showed that HCV+ adults scored significantly higher on the Somatic Factor (i.e., loss of energy, changes in sleeping pattern, irritability, changes in appetite, concentration difficulty, tiredness or fatigue, loss of interest in sex) than the Cognitive Affective Factor (i.e., sadness, pessimism, past failure, guilty feelings, punishment feelings, self-dislike, self-criticalness, suicidal thoughts, crying, agitation, worthlessness). Thus, for this study, the total BDI-II scores (Depression-Total) as well as the two BDI-II factors scores [Depression-Cognitive Affective Factor and Depression-Somatic Factor, derived according to the previously published methods (Patterson et al. 2011)] are reported and analyzed.

#### Anxiety

Generalized Anxiety Disorder Inventory (GADI; Argyropoulos et al. 2007). A well-validated 18-item measure of anxiety severity.

#### Fatigue

Fatigue Severity Scale (FSS; Krupp et al. 1989; Kleinman et al. 2000; Ferentinos et al. 2011). A nine-item fatigue scale, previously validated for use with patients with HCV, multiple sclerosis, and other chronic illnesses.

#### Pain

Brief Pain Inventory, Short Form (BPI; Cleeland and Ryan 1994; Keller et al. 2004; Tan et al. 2004). A well-validated 12-item inventory assessing both the intensity of recent pain (BPI Pain Severity [BPI-PS]) as well as the level at which it interferes with daily activities (BPI Pain Interference [BPI-PI]).

### Multiplex immune factor assessments

Following collection of the neuropsychiatric data, blood was drawn in the afternoon (mean time was 12:57 pm, SD = 01:45 h) by one-time venipuncture into cell preparation tubes (BD Vacutainer Systems, Franklin Lakes, NJ) containing 1 mL of 0.1 mol/L sodium citrate solution. The blood was then centrifuged at 1500 RCF for 20 min at room temperature (22–25°C). Plasma was separated, collected, and immediately aliquoted in polypropylene tubes (Phenix Research Products, Hayward, CA) and frozen at −80°C until assayed. Table 1 lists all factors that were assessed using Myriad Rules Based Medicine, Inc.'s (Austin, TX) Human InflammationMAP v 1.0 panel, a 47-biomarker MAP designed to discern inflammatory patterns in biological samples including plasma. Myriad Rules Based Medicine, Inc. is a Clinical Laboratory Improvement Amendments-certified laboratory. Assays conducted by this company utilizing this methodology have been published previously (e.g., Freeman et al. 2010; Schrijvers et al. 2011; Wilhelm et al. 2013). This multiplex microbead assay is based on Luminex technology (Vignali 2000) and measures proteins in a similar manner to standard sandwich ELISA, with comparable sensitivity and range. MAP assays have been compared to regular high sensitivity ELISAs in studies of Alzheimer's disease, Parkinson's disease, parasite infection, HIV, and others (e.g., O'Bryant et al. 2010 [brain-derived neurotrophic factor (BDNF)]; Codices et al. 2012 [immunoglobulins]; Camargo et al. 2009 [IL-2]; [Hu et al. 2010 (review of biomarker discovery in Alzheimer's and Parkinson's diseases)]) and produce equivalent results.

Samples were sent frozen in a single batch to Myriad Rules Based Medicine, Inc. where they were thawed for assay without additional freeze-thaw cycles. Table 1 defines each factor's abbreviation, unit of measurement, and the assay's sensitivity in terms of the least detectable concentration (LDC) (mean ± 3 standard deviations of 20 blank readings) as provided by Myriad Rules Based Medicine, Inc., and the percentage within each group with levels ≥ the LDC. For data reported above the LDC, the interassay variability was <10% for all analytes measured.

### Statistical analyses

All data analyses were conducted with SPSS, Version 17.0 (IBM Corporation, Armonk, NY) and JMP, Version 10.0 (SAS, Cary, NC). Significant *P*-values were ≤0.05 and *P*-values ≤ 0.10 were considered trends. Between-group analyses of age, education, and estimated cognitive reserve were conducted using *t*-tests; other demographic and clinical characteristics were categorical, so chi-square tests were used, or Fisher exact tests if cells had low frequencies (<5; Table 2). Mann–Whitney *U*-tests were used for between-group comparisons of neuropsychiatric symptom severity (Depression-Total, Depression-Cognitive Affective Factor, Depression-Somatic Factor, Anxiety, Fatigue, Pain Severity, and Pain Interference) because questionnaire scores (except Anxiety) were not normally distributed (Table 2). Note that in Table 2 Mann–Whitney *U*-tests were conducted on the medians. The percentages of immune factors ≥ the LDC were compared across groups with tests of two proportions, and the *z* and *P*-values are reported (Table 1). Between-group comparisons of plasma immune factor levels were computed with Mann–Whitney *U*-tests because distributions were not normal (transformations did not normalize the data), and the medians and interquartile ranges are reported (Table 1). Spearman's rank correlations were used to assess the relationship between neuropsychiatric symptom severity and the number of immune factors that were ≥ the LDC, within the total sample and by group (Table 3). On the basis of reports in the literature (e.g., Hilsabeck et al. 2010) and on Myriad Rules Based Medicine, Inc.'s customized platform used for the analyses (i.e., Human InflammationMAP® v. 1.0), an increased inflammatory profile was defined as a greater number of factors ≥ the LDC.

**Table 2:** Between-group comparisons of demographic data, clinical characteristics, and neuropsychiatric function in adults with (HCV+) and without (HCV−) hepatitis C^1^

	HCV+	HCV−	*P*-value
*n*	39	40	
Demographics			
Age, mean years (SD)^2^	52.5 (8.0)	47.9 (13.4)	0.069
Male gender	29 (76%)	29 (73%)	0.700
Caucasian	30 (77%)	28 (70%)	0.486
Veteran status	19 (49%)	20 (50%)	0.909
Years of education, mean (SD)^2^	13.8 (2.0)	13.8 (2.3)	0.951
Estimated cognitive reserve (WTAR), mean standard score (SD)^2^	101.9 (12.9)	106.8 (12.0)	0.087
Clinical characteristics			
Body mass index 2	29.8 (6.7)	28.1 (5.1)	0.191
Current tobacco use	23 (59%)	12 (30%)	0.013*
Past medical diagnoses (any)	26 (67%)	17 (43%)	0.031*
Diabetes	4 (10%)	5 (13%)	0.999
Hyperlipidemia	7 (18%)	4 (10%)	0.308
Hypertension	17 (44%)	9 (23%)	0.046*
Other cardiovascular	1 (3%)	3 (8%)	0.615
Asthma/pulmonary	12 (31%)	5 (13%)	0.048*
Current psychiatric diagnosis (any)^3^	11 (29%)	4 (10%)	0.039*
Major depressive disorder 3	4 (10%)	3 (8%)	0.712
PTSD^3^	5 (13%)	1 (3%)	0.108
Other anxiety disorder 3	6 (15%)	2 (5%)	0.154
Neuropsychiatric symptom severity			
Depression-Total (BDI-II), mean total scale score (SD)^4^	7.5 (7.2)	4.5 (5.1)	0.044*
Depression-Cognitive Affective Factor (BDI-II), mean factor score (SD)^4^	0.2 (0.4)	0.2 (0.2)	0.501
Depression-Somatic Factor (BDI-II), mean factor score (SD)^4^	0.5 (0.4)	0.3 (0.3)	0.020*
Anxiety (GADI), mean total scale score (SD)^4^	11.2 (9.8)	6.6 (7.5)	0.008**
Fatigue (FSS), mean total scale score (SD)^4^	3.6 (1.6)	2.6 (1.3)	0.006**
Pain Severity (BPI-PS), mean index score (SD)^4^	2.6 (2.4)	1.9 (2.2)	0.276
Pain Interference (BPI-PI), mean index score (SD)^4^	2.2 (2.2)	1.2 (1.8)	0.048*

BDI-II, Beck Depression Inventory-II; BPI-PI, Brief Pain Inventory-Pain Interference; BPI-PS, Brief Pain Inventory-Pain Severity; FSS, Fatigue Severity Scale; GADI, Generalized Anxiety Disorder Inventory; HCV+, adults with chronic hepatitis C virus infection; HCV−, adults with no history of infection with the hepatitis C virus; PTSD, posttraumatic stress disorder; SD, standard deviation; WTAR, Wechsler Test of Adult Reading.

1 Data expressed as *n*, with (%) in terms of *n* over total *N* unless otherwise stated. *P*-values reflect comparisons between the HCV+ group versus the HCV− control group. Chi-square was used for noncontinuous variables, or Fisher exact tests if expected cell counts were <5.

2 Student *t*-tests were used for continuous variables with normal distributions.

3 Psychiatric diagnoses were based on Diagnostic and Statistical Manual of Mental Disorders, Fourth Edition criteria verified using the Mini-International Neuropsychiatric Interview.

4 Mann–Whitney *U*-tests were used for continuous variables with nonnormal distributions.

* *P*≤ 0.050;

* *P*≤ 0.010.

**Table 3:** Bivariate correlations^1^ [*r* (*P*-values)] between number of plasma immune factors ≥ the LDC^2^ and neuropsychiatric symptom severity in adults with (HCV+) and without (HCV−) hepatitis C

	Total sample	HCV+	HCV−
Total *N*	79	39	40
Depression-Total (BDI-II), mean total scale score	0.156 (0.173)	0.258 (0.113)	−0.125 (0.448)
Depression-Cognitive Affective Factor (BDI-II), mean factor score	0.077 (0.504)	0.094 (0.568)	−0.003 (0.987)
Depression-Somatic Factor (BDI-II), mean factor score	0.202 (0.078)^*^^2001^	0.365 (0.022)^**^^2001^	−0.169 (0.310)
Anxiety (GADI), mean total scale score	0.339 (0.003)^***^^2001^	0.357 (0.028)^**^^2001^	0.075 (0.651)
Fatigue (FSS), mean total scale score	0.220 (0.057)	0.144 (0.387)	0.008 (0.964)
Pain Severity (BPI-PS), mean index score	0.169 (0.142)	0.150 (0.368)	0.120 (0.466)
Pain Interference (BPI-PI), mean index score	0.293 (0.010)^***^^2001^	0.345 (0.036)^**^^2001^	0.122 (0.461)

BDI-II, Beck Depression Inventory-II; BPI-PI, Brief Pain Inventory-Pain Interference; BPI-PS, Brief Pain Inventory-Pain Severity; FSS, Fatigue Severity Scale; GADI, Generalized Anxiety Disorder Inventory; HCV+, adults with chronic hepatitis C virus infection; HCV−, adults with no history of infection with the hepatitis C virus; LDC, least detectable concentration; SD, standard deviation.

1 Spearman's rank correlations were used to assess the relationship between neuropsychiatric symptom severity and the number of immune factors that were ≥ LDC, within the total sample and by study group. Correlations are reported with *P*-values in parentheses.

2 The LDC is defined as the mean ± 3 standard deviations of 20 blank readings as provided by Myriad Rules Based Medicine.

^*^P < 0.100; ^**^P < 0.050; ^***^P < 0.010.

Regression models were developed in order to find which combination of immune factors was significantly related to neuropsychiatric symptom severity on each of the seven neuropsychiatric variables within the total sample. Some variables had values that were undetectable. For the purpose of the analysis, these undetectable values were replaced with zeros. These undetectable values should not be confused with the LDC values used for Tables 1 through 3. Models were constructed with a backward selection linear regression of 33 immune factors (14 factors were invariant and detectable in 5% or less of the samples and were eliminated from analyses; Table 1). The backward selection started with the 33 immune factors and systematically eliminated from the model variables that were not significant (*P*-value threshold for entry *P* > 0.25, *P*-value threshold for elimination *P* > 0.10). On the basis of the final solution of the backward regression, a two-step model for each dependent variable (DV) was constructed (Table 4a–g); fit parameters are presented as well as the unstandardized regression weights (*b*), *t* values and *P*-values for each immune factor. In these models, the first step consisted of regressing the DV onto HCV status (coded 0 for the HCV− control group, and 1 for the HCV+ group). In the second step, the significant immune factors from the backward selection were entered simultaneously with HCV status to create the final model. Examination of histograms, skewness, and kurtosis values showed that the DVs in these models (except GADI) were not normal distributions. Linear regression is quite robust to deviations from normality for DVs. The impact of the nonnormality of the DVs was assessed by a plot of the predicted standardized residuals by the observed standardized residuals (P-P Plot). In all seven models, these plots showed no significant deviations from normally distributed error patterns, indicating that the nonnormality of the DV's had little to no bias on the model results. Bonferroni corrections for multiple comparisons were applied to the between-group comparisons and regression model analyses, as appropriate.

**Table 4:** Multi-analyte regression models^1^

(a) Depression-Total (BDI-II)			
Model fit	*F*(1, 77) = 4.0145;	*P* = 0.0487;	*R*^2^ = 0.0502
Variable	*b*	*t*	*P*
Intercept	1.8205	0.80	0.4284
HCV status	2.8974	2.00	0.0487
Model fit	*F*(8, 77) = 4.787;	*P* < 0.001;	*R*^2^ = 0.3567
Variable	*b*	*t*	*P*
Intercept	17.3226	4.20	<0.0001
HCV status	5.1596	3.23	0.0019
A2Macro	−12.1283	−2.02	0.0475
BDNF	−1.4825	−2.89	0.0052
Eotaxin1	−0.0165	−3.15	0.0024
IL23	4.7228	2.96	0.0042
RANTES	0.4548	1.99	0.0509
TNF*α*	2.6492	3.32	0.0015
TNFR2	−3.1421	−4.29	<0.0001
(b) Depression-Cognitive Affective Factor (BDI-II)			
Model fit	*F*(1, 77) = 1.364;	*P* = 0.2466;	*R*^2^ = 0.0176
Variable	*b*	*t*	*P*
Intercept	0.0862	0.80	0.4241
HCV status	0.0793	1.17	0.2466
Model fit	*F*(11, 77) = 4.0268;	*P* < 0.0002;	*R*^2^ = 0.4016
Variable	*b*	*t*	*P*
Intercept	0.2661	1.35	0.1831
HCV status	0.1556	2.12	0.0375
AAT	0.4588	2.52	0.0143
BDNF	−0.0731	−3.11	0.0028
Eotaxin1	−0.0005	−2.03	0.0465
IL15	−0.1373	−2.05	0.0441
IL18	−0.0011	−1.67	0.0997
IL23	0.2184	3.00	0.0039
RANTES	0.0221	2.10	0.0394
TNF*α*	0.1198	3.22	0.0020
TNFR2	−0.2101	−4.96	<0.0001
vWF	0.0088	1.93	0.0579
(c) Depression–Somatic Factor (BDI-II)			
Model fit	*F*(1/76) = 6.1293,	*P* = 0.0156,	*R*^2^ = 0.0756
Variable	*b*	*t*	*P*
Intercept	0.1001	0.75	0.4559
HCV status	0.2082	2.48	0.0156
Model fit	*F*(9/76) = 3.2644	*P* = 0.0024,	*R*^2^ = 0.3048
Variable	*b*	*t*	*P*
Intercept	1.0215	3.25	0.0018
HCV status	0.3342	3.49	0.0009
A2Macro	−0.9127	−2.39	0.0196
C3	1.1813	1.75	0.0841
Fibrinogen	−0.1525	−1.88	0.0649
IL23	0.2075	2.07	0.0426
IL8	0.0146	1.95	0.0558
MCP1	−0.0031	−2.56	0.0128
MIP1*β*	−0.0020	−2.57	0.0123
MMP3	−0.0538	−2.14	0.0363
(d) Anxiety (GADI)			
Model fit	*F*(1, 76) = 5.4028;	*P* < 0.0228;	*R*^2^ = 0.0672
Variable	*b*	*t*	*P*
Intercept	2.0202	0.65	0.5184
HCV status	4.5951	2.32	0.0228
Model fit	*F*(5, 76) = 4.7444;	*P* < 0.009;	*R*^2^ = 0.2504
Variable	*b*	*t*	*P*
Intercept	0.6811	0.17	0.8628
HCV status	4.9587	2.13	0.0370
MIP1*α*	0.1114	1.68	0.0966
SCF	0.0487	1.92	0.0589
TNF*α*	2.3309	1.98	0.0516
TNFR2	−3.1440	−3.20	0.0020
(e) Fatigue (FSS)			
Model fit	*F*(1, 75) = 9.0997;	*P* < 0.035;	*R*^2^ = 0.1095
Variable	*b*	*t*	*P*
Intercept	1.6061	3.06	0.0030
HCV status	1.0000	3.02	0.0035
Model fit	*F*(8, 75) = 4.3840;	*P* < 0.0003;	*R*^2^ = 0.3436
Variable	*b*	*t*	*P*
Intercept	0.7798	0.78	0.4359
HCV status	0.5099	1.46	0.1485
AAT	2.1043	2.17	0.0335
BDNF	−0.2359	−2.10	0.0396
FactorVII	0.0019	1.93	0.0583
IL7	0.1587	3.17	0.0023
RANTES	0.1255	2.38	0.0200
VDBP	−0.0042	−1.99	0.0511
VEGF	−0.0076	−2.11	0.0385
(f) Pain Severity (BPI-PS)			
Model fit	*F*(1, 76) = 1.4521;	*P* < 0.2320;	*R*^2^ = 0.0190
Variable	*b*	*t*	*P*
Intercept	1.2919	1.55	0.1253
HCV status	0.6376	1.21	0.2320
Model fit	*F*(5, 76) = 3.2423;	*P* < 0.0108;	*R*^2^ = 0.1859
Variable	*b*	*t*	*P*
Intercept	0.9130	0.81	0.4195
HCV status	0.7797	1.47	0.1468
IL10	−0.2597	−1.82	0.0722
IL5	0.0642	2.26	0.0267
MIP1*β*	−0.0111	−2.28	0.0255
SCF	0.0148	2.18	0.0325
(g) Pain Interference (BPI-PI)			
Model fit	*F*(1, 75) = 4.1609;	*P* < 0.0449;	*R* = 0.0532
Variable	*b*	*t*	*P*
Intercept	0.2677	0.37	0.7155
HCV status	0.9513	2.04	0.0449
Model fit	*F*(5, 75) = 3.5498;	*P* < 0.0064;	*R*^2^ = 0.2023
Variable	*b*	*t*	*P*
Intercept	0.6042	0.71	0.4774
HCV status	1.0427	2.16	0.0338
CRP	0.2380	1.80	0.0755
IL10	−0.2695	−2.04	0.0456
MMP3	−0.3068	−2.19	0.0316
TNF*α*	0.5051	1.93	0.0574

BDI-II, Beck Depression Inventory-II; BPI-PI, Brief Pain Inventory-Pain Interference; BPI-PS, Brief Pain Inventory-Pain Severity; BDNF, brain-derived neurotrophic factor, CRP, C-reactive protein; DV, dependent variable; FSS, Fatigue Severity Scale; GADI, Generalized Anxiety Disorder Inventory; HCV+, adults with chronic hepatitis C virus infection; HCV−, Adults with no history of infection with the hepatitis C virus; MIP, macrophage inflammatory protein; RANTES, Regulated upon Activation, Normal T-cell Expressed, and Secreted; TNF, tumor necrosis factor; TNFR, Tumor Necrosis Factor Receptor 2; vWF, von Willebrand factor.

1 Regression models were developed in order to find which combination of plasma immune factors was significantly predictive of neuropsychiatric symptom severity on each of the seven neuropsychiatric variables within the total sample. Models were constructed with a backward selection linear regression of 33 immune factors. The backward selection started with the 33 immune factors and systematically eliminated from the model variables that were not significant, retaining only those with *P*-values (*P* ≤ 0.10). HCV Status was not allowed to be eliminated. Based on the final solution of the backward regression, a two-step model for each neuropsychiatric variable (DV) was constructed and these are presented above; fit parameters are presented as well as the unstandardized regression weights (*b*), *t* values and *P*-values for each immune factor. In these models, the first step consisted of regressing the DV onto HCV status (coded 0 for the HCV− control HCV Status, and 1 for the HCV+ HCV Status). In the second step, the significant immune factors from the backward selection were entered simultaneously with HCV status to create the final model. See Table 1 for immune factor abbreviations.

## Results

### Demographic data, clinical characteristics, and neuropsychiatric function

Within the HCV+ group of participants, 66.7% (*n* = 26) reported contracting HCV through intravenous drug use, 7.7% (*n* = 3) through tattoos, 5.1% (*n* = 2) through accidental work exposures, 2.6% (*n* = 1) through blood transfusions, and 17.9% (*n* = 7) through unknown or other unspecified causes. HCV disease characteristics for the HCV+ group are as follows (reported as mean values ± standard deviation): HCV RNA (log10 IU/mL) = 5.9 ± 0.9, serum aspartate aminotransferase levels (AST) = 55.7 ± 41.8, alanine aminotransferase levels (ALT) = 78.3 ± 54.9, and platelet levels = 221.2 ± 78.8. 82% (32/39) of participants had HCV genotypes available in their records (53% [17/32] with genotype 1, 22% [7/32] with genotype 2, and 25% [8/32] with genotype 3).

Table 2 summarizes demographic data and clinical characteristics by study group. Groups did not significantly differ in terms of age, gender, race, veteran status, years of education, estimated cognitive reserve as measured by the Wechsler Adult Reading Test (Wechsler 2001), or body mass index. HCV+ adults were more likely to currently use tobacco products than the HCV− controls. Although adults with currently severe or unstable medical conditions were excluded from participation, HCV+ adults were more likely than controls to have a history of any medical condition other than HCV, and a history of hypertension or asthma in particular. Although adults with psychotic disorders or currently severe or unstable psychiatric disorders were excluded from participation, HCV+ adults were more likely than controls to have any current psychiatric diagnosis (major depressive disorder, PTSD, or other anxiety disorders). Table 2 shows that HCV+ adults also reported significantly greater neuropsychiatric symptom severity on measures of depression (Depression-Total and Depression-Cognitive Affective Factor), anxiety, fatigue, and pain (Pain Interference) than controls.

### Between-group comparisons of plasma immune factors

Table 1 summarizes the results of between-group comparisons of plasma immune factor profiles. Relative to HCV− controls, HCV+ adults had significantly higher plasma levels of 40% (19/47) of the immune factors. Compared with the HCV+ group, the HCV− group had significantly higher plasma levels of one immune factor (i.e., C-reactive protein). Following a Bonferroni correction for multiple comparisons, 21% (10/47) of the immune factors (i.e., *α*-2-macroglobulin [A2Macro], *β*-2-microglobulin [B2M], intracellular adhesion molecule [ICAM]-1, IL-18, IL-8, macrophage inflammatory protein [MIP]-1*α*, tissue inhibitor of metalloproteinases [TIMP]-1, tumor necrosis factor receptor [TNFR]2, vascular cell adhesion molecule-1 [VCAM-1], and von Willebrand factor [vWF]) remained significantly different between groups using a Bonferroni cutoff of *P* = 0.001 (i.e., 0.05/47 between-group comparisons). Relative to HCV− controls, HCV+ adults had a significantly higher percentage of individuals with plasma immune factor levels ≥ the LDC for three of the immune factors (i.e., IL-10, MIP-1*α*, TNF-*α*); these differences did not remain significant after a Bonferroni correction with a cutoff of *P* = 0.001.

### Immune factor correlates of neuropsychiatric symptom severity

Table 3 summarizes correlations between the number of plasma immune factors ≥ the LDC and neuropsychiatric symptom severity within the total sample and each study group. Within the total sample, an increased inflammatory profile, as indicated by higher numbers of immune factors ≥ the LDC, significantly correlated with Anxiety and Pain Interference, and it trended toward significance for Depression-Somatic Factor. The correlations with Depression-Somatic Factor, Anxiety, and Pain Interference were significant in the HCV+ group alone, but not in the HCV− control group alone.

In order to evaluate the possibility that an increased inflammatory profile was a proxy for common HCV disease severity markers, we conducted post hoc correlations (Spearman's rank) within the HCV+ group between number of immune factors ≥ the LDC and HCV viral load (HCV RNA), AST levels, and ALT levels; none of these HCV disease severity markers significantly correlated with number of immune factors ≥ the LDC (data not shown), suggesting that the inflammatory profile was independent from other HCV disease severity markers.

In Table 4, each of the regression models had a single Type I error rate for the predictors that was determined by the omnibus test of the model fit, limiting the risk of Type I error due to multiple comparisons. Fourteen regression models (i.e., seven DVs each with two tests run, one model with HCV status entered as a predictor on its own, and a second model with HCV status and the immune factors entered as predictors together) were calculated. A Bonferroni correction with a cutoff of *P* = 0.0035 (i.e., 0.05/14 tests) determined if the models were significant after a correction. Thus, the *P*-value for the omnibus model must be below this cutoff for models to be significant; individual variable *P*-values are considered significant if <0.05 as long as the model is significant. For the models with only HCV Status entered, only the FSS was significant. For the models with multiple analytes entered, the significant models were for Depression Total, Depression-Cognitive Affective, Depression-Somatic, Anxiety, and Fatigue. The two pain scales had *P*-values above this threshold. Results from these models are interpreted cautiously.

Ignoring the correction briefly, as summarized in Table 4, and consistent with the group comparisons in Table 2, HCV status was a significant predictor of increased Depression-Total, Depression-Somatic Factor, Anxiety, Fatigue, and Pain Interference in regression analyses with HCV status entered as the only independent variable. Depression-Cognitive Affective and Pain Severity were not significant in either Tables 2012 or 2000. Therefore, HCV status was entered as an independent variable along with the 33 detectable immune factors in subsequent regression analyses. In the final regression models (Table 4), HCV status was a significant predictor of the severity of Depression-Total, Depression-Cognitive Affective Factor, Depression-Somatic Factor, and Anxiety. All of the final regression models accounted for a larger percentage of the variance in each neuropsychiatric variable than HCV status alone. The final regression models yielded protein signatures of 4–10 plasma immune factors that significantly predicted the severity of each neuropsychiatric variable. The protein signatures accounted for 36% of the variance in Depression-Total (seven factors), 40% in Depression-Cognitive Affective Factor (10 factors), 31% in Depression-Somatic Factor (eight factors), 25% in Anxiety (four factors), and 34% in Fatigue (seven factors). Results were interpreted cautiously because models were significant, but not at the corrected level for Pain Severity (19%; four factors) and Pain Interference (20%; four factors).

Because of extant group differences in rates of hypertension, asthma, and current tobacco use (Table 2), we evaluated whether HCV was a proxy that would account for differences in our models (Sluzewska et al. 1995; Maes et al. 1999, 2009; Kubera et al. 2001; O'Brien et al. 2007). To do this, we conducted linear regression analyses to evaluate these variables as potential covariates in our models. When each of these variables was entered as sole predictors of neuropsychiatric symptom severity in regression models, they were each significant (data not shown); however, when entered into the models as predictors along with HCV disease status, none of these variables remained significant (data not shown). Moreover, entering these variables along with HCV status and the immune factors into the regression models did not alter the final models (i.e., all immune factors found to be significant previously, remained significant). In short, these variables appeared to be weak proxies for HCV status within the regression models and were not deemed significant covariates. Inclusion of HCV status in Table 4 accounts fully for these differences.

### Exploratory analyses

Although alcohol or drug dependence within the past year (except nicotine or caffeine) was an exclusionary criterion for this study, it is possible that a remote history of substance dependence may be associated with more persistent effects on neuropsychiatric symptoms and immune factor expression (e.g., Wang et al. 2004; Sekine et al. 2008; Potter et al. 2013) and may therefore affect the composition of the multi-analyte regression models. Based on a chi-square test, a significantly (*P* < 0.001) greater percentage of adults in the HCV+ group (76.9%) met DSM-IV criteria, based on the MINI, for a lifetime history of dependence on alcohol or other drugs compared with the HCV− group (35.0%). The history of substance dependence was notably remote for both groups; there were no significant differences across groups in terms of mean length of remission from all substances (HCV+ = 7.7 years; HCV− = 8.8 years; *P* = 0.649). The percentage of adults within each group who met DSM-IV criteria for lifetime dependence, based on the MINI, for specific substances are as follows: alcohol (HCV+ = 51.3%; HCV− = 27.5%; *P* = 0.030), stimulants (HCV+ = 56.4%; HCV− = 25.0%; *P* = 0.004), marijuana (HCV+ = 28.2%; HCV− = 10.0%; *P* = 0.039), opiates (HCV+ = 38.5%; HCV− = 2.5%; *P* < 0.001), and other drugs (HCV+ = 10.3%; HCV− = 2.5%; *P* = 0.201); note that these groups are not mutually exclusive because many participants had a lifetime history of polysubstance dependence (HCV+ = 64.1%; HCV− = 22.5%; *P* < 0.001). History of intravenous drug use was not recorded, except in the HCV+ group if that was how HCV was reportedly contracted. Exploratory analyses to evaluate the impact of any substance dependence history on neuropsychiatric symptom immune factor profiles generally yielded regression models that were similar to the models shown in Table 4 (see Table S1). In this analysis, the first models added HCV status and an indicator from the MINI of any alcohol or drug dependence diagnosis. For the last section, these variables were entered with 33 immune factors and were locked to elimination in the backwards regression selection. The final models were simultaneous regressions with the remaining variables entered. Noteworthy differences between Tables 2000 and S1 include: (1) the addition of B2M to models describing the cognitive-affective depression factor, somatic depression factor, and anxiety factor, (2) the addition of TNFR2 to the somatic depression factor, (3) the addition of IL23 to the anxiety factor, and (4) the removal of TNF-*α* from the pain interference factor.

## Discussion

Overall, results indicate that, compared with noninfected and demographically similar HCV− controls, treatment naïve HCV+ adults present with increased neuropsychiatric symptoms including aspects of depression (somatic symptoms), anxiety, fatigue, and pain (pain interference). Similar to previous studies, our data (Table 1) indicate that, compared to adults without HCV, adults with HCV have higher plasma levels of *α*-2-macroglobulin (A2Macro; Ho et al. 2010), *β*-2-microglobulin (B2M; Malaguarnera et al. 2000; ŁApiński et al. 2002), ICAM-1 (El-Gohary et al. 2004; Helaly and Abou Shamaa 2006), IL-8 (Zimmermann et al. 2011; Sousa et al. 2012; Warshow et al. 2012), IL-18 (Sharma et al. 2009; Wilkinson et al. 2010; Akcam et al. 2012), MIP-1*α* (Larrubia et al. 2008; Florholmen et al. 2011), tissue inhibitor of metalloproteinases (TIMP)-1 (Leroy et al. 2004), TNFR2 (Pawlak et al. 2010), vascular cell adhesion molecule-1 (VCAM-1; Bruno et al. 2005; Pawlak et al. 2010), and vWF (Pawlak et al. 2010); these group differences remained significant following a Bonferroni correction for multiple comparisons across an array of 47 immune factors, highlighting the robustness of these findings. Moreover, HCV+ adults are more likely than controls to have an increased inflammatory profile. Within the HCV+ group, but not within the HCV− group, number of inflammatory factors with levels ≥ the LDC significantly correlated with several neuropsychiatric symptoms, showing that an HCV-associated increased inflammatory profile is associated with increased neuropsychiatric symptom severity, specifically aspects of depression (somatic symptoms), anxiety, and pain (pain interference).

Results additionally suggest that differences in expression of the network of peripheral immune proteins significantly impact neuropsychiatric function in adults, regardless of HCV status. Neuropsychiatric symptom severity was significantly predicted by specific protein signatures, consisting of 4–10 plasma immune factors depending on the neuropsychiatric variable, after controlling for HCV status. Each panel of significant immune factors accounted for 19–40% of the variance in depression, anxiety, fatigue, and pain. These analyses reveal potential disease signatures and individually significant immune factors worthy of further investigation through confirmatory studies (e.g., as treatment targets).

**Figure 1:**
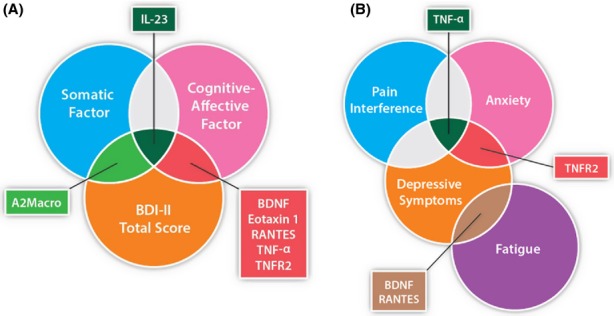
Venn diagrams of differentially expressed proteins (biomarkers) illustrate the common and shared inflammatory factors associated with depressive (A) and neuropsychiatric (B) symptom severities.

A major goal of this study was to identify novel biomarkers that might be relevant to the discovery and development of new treatments for neuropsychiatric symptoms. Five proteins were related to more than one neuropsychiatric variable and are of interest for future study—BDNF, IL-23, RANTES, TNF-*α*, and TNFR2 (Fig. 1). Because these five biomarkers may be most relevant to neuropsychiatric symptoms, and because it is beyond the scope and length of this article to discuss each of the significant immune factors identified in our regression analyses, the following discussion is focused on these five factors, all of which have both immunoregulatory as well as neuromodulatory functions.

### BDNF

Alterations in neurogenesis and neuronal plasticity are observed in a number of CNS disorders that contain inflammatory processes. BDNF (a member of the neurotrophic factor family) is implicated as a key mediator of this plasticity, and inflammatory cytokines (e.g., IL-1*β*) can decrease BDNF signaling (Tong et al. 2008; Cortese et al. 2011). Regulation of BDNF expression and function contributes, in part, to the pathophysiology and treatment of depression (Chen et al. 2001; Sen et al. 2008). Both the Val66Met BDNF polymorphism (rs6265) and BDNF levels have been associated with depression (Egan et al. 2003; Hashimoto 2010). BDNF levels also correlate with treatment outcomes, and may, therefore, be a useful biomarker for prognosis (Kurita et al. 2012). Importantly, for patients with HCV, BDNF levels appear to influence resiliency against developing depression during interferon-*α*-based therapies (Lotrich et al. 2012).

### IL-23

IL-23 is an important mediator of the inflammatory response against infection. In conjunction with IL-6 and transforming growth factor (TGF)-*β* 1, IL-23 stimulates naive CD4+ T cells to differentiate into Th17 cells (T-cell subsets that produce IL-17, a proinflammatory cytokine that can stimulate the production of other proinflammatory factors, such as IL-1, IL-6, and TNF-*α*; Kikly et al. 2006; Langowski et al. 2006). Although little is known about its role in brain and effect on neuropsychiatric function, inhibition of the IL-12/IL-23 pathway reduces microglia activation and improves cognitive function and related pathology in an Alzheimer's disease mouse model (Vom Berg et al. 2012). Similarly, knockout mice deficient in either IL-23 subunits p40 or p19, or in either subunit of the IL-23 receptor (IL-23R and IL12R-*β*1) develop less severe symptoms of multiple sclerosis and inflammatory bowel disease (Gran et al. 2004; Yen et al. 2006). Consistent with these observations, we found that increased plasma IL-23 concentrations were associated with increased depression severity ratings (Table 4; Fig. 1); however, more research is needed to investigate the role of IL-23 signaling in CNS inflammatory diseases, including depression.

### RANTES

Regulated upon Activation, Normal T-cell Expressed, and Secreted (a.k.a. chemokine [C-C motif] ligand 5 [CCL5]) is a potent chemoattractant for T lymphocytes and mononuclear phagocytes and plays an active role in recruiting leukocytes into inflammatory sites (Schall and Bacon 1994). Tawadrous et al. (2012) found significant increases in the levels of RANTES (as well as TNF-*α* and other inflammatory factors) in patients with HCV compared to patients without HCV. Furthermore, in this study RANTES levels showed a significant positive correlation with HCV RNA viral loads; however, mood and other neuropsychiatric symptoms were not assessed. In other clinical studies, RANTES is included among the biomarkers associated with Alzheimer's disease, mild cognitive impairment (Marksteiner et al. 2011), and hostility (Mommersteeg et al. 2008). Although a direct association between RANTES and depression has yet to be established, Mommersteeg et al. (2008) found that early-life trauma and depression were positively and independently related to hostility.

### TNF-*α* and TNFR2

Tumor necrosis factor-*α* is a proinflammatory cytokine [recently described as a neuroactive cytokine (Jones and Thomsen 2013)] that is released following immune challenges, stimulating the release of additional immune factors. TNF-*α* has been linked with neuropsychiatric symptoms, particularly depression in a number of studies (e.g., Himmerich et al. 2008; Dowlati et al. 2010; Duivis et al. 2013; Loftis et al. 2013a). Blockade of TNF-*α* is being evaluated both preclinically and clinically as a possible treatment for depression, and levels of TNF-*α* may also help predict antidepressant treatment response (Rethorst et al. 2013; Krügel et al. 2013; Raison et al. 2013).

Tumor necrosis factor-*α* binds to one of two receptors, TNFR1 and TNFR2 (Schafers et al. 2008). Elevated blood levels of TNFR2 are found in patients with major depressive disorder compared with nondepressed controls (Grassi-Oliveira et al. 2009; Diniz et al. 2010), and TNFR2 levels correlate with depression severity in depressed patients (Grassi-Oliveira et al. 2009). Compared with wild-type mice, TNFR1- and TNFR2-deficient mice evidence reduced depression-like (Simen et al. 2006) and anxiety-like (Patel et al. 2010) behaviors, providing additional support for the putative link between depression and anxiety disorders and inflammation (Miller et al. 2013; Fig. 1). Although TNFR2 was not significantly predictive of pain in this study, TNFR1- and TNFR2-deficient mice have been shown to exhibit reduced pain responses (Vogel et al. 2006). TNF-*α* is believed to sensitize primary afferent nerves and to therefore increase pain responses to additional stimuli through TNFR1 and TNFR2 signaling (Schafers et al. 2008). Our results indicate that it may be of interest to evaluate whether, in the context of chronic HCV, TNF-*α* and TNFR2 signaling could similarly contribute toward the sensitization of neurons in a manner that enhances other neuropsychiatric symptoms (e.g., depression, anxiety, and fatigue).

The identification of disease-specific combinations (i.e., signatures) of blood proteins may lead to recognition of specific risk patterns relevant to patient outcomes, tools for tracking treatment progress, or the identification of potential treatment targets or strategies; indeed, through analysis of large arrays, unique protein signatures have been associated with markers of a wide range of conditions including cancer (Lee et al. 2009; Chen et al. 2010), HIV-related cognitive impairment (Toro-Nieves et al. 2009), dementia (Gomez Ravetti and Moscato 2008; Britschgi and Wyss-Coray 2009), multiple sclerosis (De Masi et al. 2009), pain disorders (Slade et al. 2011; Alexander et al. 2012), and psychiatric disorders, including depression (Simon et al. 2008; Domenici et al. 2010; Arnold et al. 2012). Similarly, the results from this study strongly demonstrate the degree to which immune cell signaling proteins influence neuropsychiatric function in adults with and without HCV, and they suggest that efforts to develop and investigate novel immunotherapies as treatments for neuropsychiatric symptoms are warranted. As described above, our results show that BDNF, IL-23, RANTES, TNF-*α*, and TNFR2 may be of particular relevance to HCV-associated neuropsychiatric symptoms. Like many signaling proteins, these factors have dual roles as immunoregulators and neuromodulators, with the potential to both enhance inflammatory responses as well as adversely impact neuronal functions (e.g., target neurons for cell death, alter synaptic plasticity, hamper neuronal repair, enhance sensitivity to pain or other stimuli) when upregulated. Thus, immunotherapies that are designed to simultaneously “normalize” immunoregulation and neuromodulation may be particularly effective in treating neuropsychiatric symptoms, especially in individuals with chronic inflammatory conditions or infections such as HCV.

Consistent with this approach, neurotransmitter-based antidepressants appear to have at least indirect anti-inflammatory effects and may partially reverse derangements in relevant inflammatory factors (Maes et al. 2009). Fluoxetine treatment for depression reduces serum IL-6 in patients (Sluzewska et al. 1995), and imipramine, clomipramine, venlafaxine, fluoxetine, sertraline, and trazodone have been shown to reduce the IFN-gamma/IL-10 ratio of human blood samples (a ratio of proinflammatory/anti-inflammatory drive), consistent with an anti-inflammatory effect (Sluzewska et al. 1995; Maes et al. 1999; Kubera et al. 2001). In addition, nonresponders to selective serotonin reuptake inhibitor medication continue to exhibit elevated IL-6 levels, raising the possibility that response to treatment is linked to a reduction in IL-6 (O'Brien et al. 2007). Thus, immunotherapies that more directly target immune cell signaling may prove efficacious as primary or adjunct treatments for neuropsychiatric disorders. Clinical trials have already demonstrated the antidepressant benefits of three immunotherapies, etanercept (TNF-*α* antagonist used to treat a range of autoimmune conditions including psoriasis and arthritis), infliximab (monoclonal antibody against TNF-*α* also used for the treatment of autoimmune diseases), and celecoxib (cyclooxygenase-2 inhibitor used to treat pain and arthritis; Muller et al. 2006; Tyring et al. 2006; Raison et al. 2013). Additional immunotherapies for the treatment of neuropsychiatric disorders are currently under investigation by our lab and others (e.g., Loftis et al. 2013b).

## Strengths and Limitations

This study includes both strengths and limitations. Current substance abuse was evaluated using self-report measures (rather than drug testing). Although self-reports were cross-validated by study personnel who reviewed each participant's medical record to assess substance use history and current use patterns, it is possible that some participants may have had ongoing substance abuse. Prior use of injectable drugs may have contributed to the altered expression of immune factors in adults with HCV (or without HCV). Some studies show that injection drug use—currently, the most common way to become infected with HCV—is also associated with increased levels of proinflammatory cytokines (e.g., Graham et al. 2007). Given that history of intravenous drug use was not recorded in this study (except in the HCV+ group if that was how HCV was reportedly contracted), we are not able to determine whether a remote history of injection drug use impacted the inflammatory profiles. Furthermore, a cross-sectional study design does not allow for definitive conclusions on causality, and regression analyses are considered exploratory in nature. The models we used were constructed with backward regression for the purpose of finding an optimal set of models that significantly predict neuropsychiatric symptom severity. Such an approach does carry with it a risk of developing models that are data set specific. Therefore, future research is necessary to cross-validate the protein signatures in additional samples. Protein signatures need to be evaluated with reference to population norms in nationally representative samples prior to clinical application (e.g., for diagnostic purposes or to track symptom severity or treatment progress), as Luminex-based platforms can vary in their ability to measure serum and/or plasma concentrations of cytokines. However, these multiplex assays generally detect similar patterns of cytokine alterations and may be useful for studies in which relative, rather than absolute, changes in cytokines are of interest (Breen et al. 2011).

## Conclusions and Clinical Implications

Despite limitations, through MAP analysis of 47 plasma immune factors, this study demonstrates that adults with HCV evidence increased peripheral immune activation and increased expression of immune-related proteins that are associated with a constellation of neuropsychiatric symptoms. Moreover, results suggest that altered expression of a network of plasma immune factors contributes to neuropsychiatric symptom severity (i.e., depression, anxiety, fatigue, pain) in adults with and without HCV. This study identifies several immune factors, including BDNF, IL-23, RANTES, TNF-*α*, and TNFR2 that may be particularly relevant to neuropsychiatric symptoms and which regulate both immune and neuronal functions.

## Supplementary Information

Table S1 Multi-analyte regression models, including any history of substance dependence as a variable.
