# Supplementary material for: Multi-analyte profile analysis of plasma immune proteins: altered expression of peripheral immune factors is associated with neuropsychiatric symptom severity in adults with and without chronic hepatitis C virus infection
Source: Brain Behav. 2013 Dec 29;4(2):123–42. doi: 10.1002/brb3.200 (PMC3967530; doi:10.1002/brb3.200)
Supplement: Table S1 — Multi-analyte regression models, including any history of substance dependence as a variable. [file brb30004-0123-sd1.docx]

**Supplementary Information**

**Table 1**: **Multi-analyte regression models, including any history of substance dependence as a variable.**

| **A. Depression-Total (BDI-II)** | | | |  |
| --- | --- | --- | --- | --- |
| **Model Fit** | **F (2, 75) = 4.4380; p = 0.0151; R^2^ = 0.1058** | | |  |
| **Variable** | **b** | **t** | **p** |  |
| Intercept | 2.1705 | 0.97 | 0.3355 |  |
| HCV Status | 1.4098 | 0.90 | 0.3725 |  |
| Any Substance Dependence | 3.4128 | 2.16 | 0.0339 |  |
| **Model Fit** | **F (9, 77) = 4.8192; p < 0.001; R^2^ = .3894** | | |  |
| **Variable** | **b** | **t** | **p** |  |
| Intercept | 17.0620 | 4.22 | <.0001 |  |
| HCV status | 3.5541 | 2.00 | 0.0496 |  |
| Any Substance Dependence | 2.7461 | 1.91 | 0.0603 |  |
| A2Macro | -11.1926 | -1.89 | 0.0629 |  |
| BDNF | -1.5428 | -3.05 | 0.0032 |  |
| Eotaxin1 | -0.0154 | -2.96 | 0.0042 |  |
| IL23 | 4.5905 | 2.93 | 0.0046 |  |
| RANTES | 0.4611 | 2.05 | 0.0439 |  |
| TNFalpha | 2.7378 | 3.49 | 0.0009 |  |
| TNFR2 | -2.9639 | -4.09 | 0.0001 |  |
| **B. Depression– Cognitive Affective Factor (BDI-II)** | | | |  |
| **Model Fit** | **F (2, 77) = 1.6097; p = 0.2068; R^2^ = 0.0412** | | |  |
| **Variable** | **b** | **t** | **p** |  |
| Intercept | 0.0968 | 0.90 | 0.3688 |  |
| HCV Status | 0.0346 | 0.46 | 0.6462 |  |
| Any Substance Dependence | 0.1024 | 1.36 | 0.1789 |  |
| **Model Fit** | **F (11, 77) = 3.5151; p < 0.0005; R^2^ = 0.3936** | | |  |
| **Variable** | **b** | **t** | **p** |  |
| Intercept | 0.4674 | 2.00 | 0.0496 |  |
| HCV status | 0.0292 | 0.32 | 0.7491 |  |
| Any Substance Dependence | 0.0848 | 1.21 | 0.2306 |  |
| A2Macro | -0.5290 | -1.79 | 0.0783 |  |
| AAT | 0.4309 | 2.29 | 0.0255 |  |
| B2M | 0.4649 | 1.87 | 0.0661 |  |
| BDNF | -0.0706 | -2.96 | 0.0043 |  |
| CRP | -0.0375 | -1.81 | 0.0752 |  |
| Eotaxin1 | -0.0007 | -2.84 | 0.0060 |  |
| IL23 | 0.2650 | 3.54 | 0.0008 |  |
| RANTES | 0.0206 | 1.95 | 0.0554 |  |
| TNFalpha | 0.1171 | 3.09 | 0.0029 |  |
| TNFR2 | -0.2202 | -4.54 | <.0001 |  |
| **C. Depression–Somatic Factor (BDI-II)** | | | |  |
| **Model Fit** | **F (1, 76) = 5.0879, p = .0085, R^2^ = .1209** | | |  |
| **Variable** | **b** | **t** | **p** |  |
| Intercept | 0.1154 | 0.88 | 0.3825 |  |
| HCV Status | 0.1314 | 1.44 | 0.1550 |  |
| Any Substance Dependence | 0.1799 | 1.95 | 0.0545 |  |
| **Model Fit** | **F (9, 76) = 3.6787 p = .0009, R^2^ = .3307** | | |  |
| **Variable** | **b** | **t** | **p** |  |
| Intercept | 0.7971 | 3.39 | 0.0012 |  |
| HCV status | 0.1445 | 1.27 | 0.2068 |  |
| Any Substance Dependence | 0.2066 | 2.27 | 0.0262 |  |
| A2Macro | -0.8099 | -2.13 | 0.0372 |  |
| B2M | 0.7057 | 2.35 | 0.0219 |  |
| IL23 | 0.1696 | 1.77 | 0.0819 |  |
| IL8 | 0.0193 | 2.45 | 0.0169 |  |
| MCP1 | -0.0034 | -2.83 | 0.0061 |  |
| MMP3 | -0.0543 | -2.10 | 0.0399 |  |
| TNFR2 | -0.1551 | -2.58 | 0.0121 |  |
| **D. Anxiety (GADI)** | | | |  |
| **Model Fit** | **F (2, 76) = 6.7432 p < 0.0020; R^2^ = 0.1541** | | |  |
| **Variable** | **b** | **t** | **p** |  |
| Intercept | 2.2807 | 0.76 | 0.4475 |  |
| HCV Status | 2.2664 | 1.09 | 0.2782 |  |
| Any Substance Dependence | 5.7616 | 2.76 | 0.0073 |  |
| **Model Fit** | **F (8, 76) = 4.9902; p < 0.0001; R^2^ = 0.3699** | | |  |
| Intercept | 0.3293 | 0.08 | 0.9402 |  |
| HCV status | 1.7448 | 0.73 | 0.4676 |  |
| Any Substance Dependence | 6.4026 | 3.19 | 0.0021 |  |
| B2M | 14.1682 | 2.13 | 0.0371 |  |
| IL23 | 4.3913 | 2.07 | 0.0423 |  |
| SCF | 0.0499 | 2.08 | 0.0413 |  |
| TNFalpha | 3.0221 | 2.76 | 0.0073 |  |
| TNFR2 | -4.8020 | -3.47 | 0.0009 |  |
| VEGF | -0.0311 | -1.74 | 0.0857 |  |
| **E. Fatigue (FSS)** | | | |  |
| **Model Fit** | **F (2, 75) = 5.8095; p < 0.046; R^2^ = 0.1373** | | |  |
| **Variable** | **b** | **t** | **P** |  |
| Intercept | 1.6502 | 3.17 | 0.0022 |  |
| HCV Status | 0.7645 | 2.11 | 0.0384 |  |
| Any Substance Dependence | 0.5594 | 1.53 | 0.1294 |  |
| **Model Fit** | **F (9, 75) = 4.9630; p < 0.0001; R^2^ = 0.4036** | | |  |
| **Variable** | **b** | **t** | **P** |  |
| Intercept | 1.1792 | 1.22 | 0.2275 |  |
| HCV Satus | 0.1091 | 0.30 | 0.7686 |  |
| Any Substance Dependence | 0.8761 | 2.58 | 0.0122 |  |
| AAT | 1.8223 | 1.94 | 0.0562 |  |
| BDNF | -0.3009 | -2.71 | 0.0085 |  |
| FactorVII | 0.0021 | 2.22 | 0.0297 |  |
| IL7 | 0.1812 | 3.71 | 0.0004 |  |
| RANTES | 0.1347 | 2.66 | 0.0099 |  |
| VDBP | -0.0041 | -2.03 | 0.0459 |  |
| VEGF | -0.0074 | -2.12 | 0.0374 |  |
| **F. Pain Severity (BPI-PS)** | | | |  |
| **Model Fit** | **F (2, 76) = 2.4645; p < 0.0920; R^2^ = 0.0624** | | |  |
| **Variable** | **b** | **t** | **p** |  |
| Intercept | 1.3399 | 1.63 | 0.1067 |  |
| HCV Status | 0.2080 | 0.36 | 0.7163 |  |
| Any Substance Dependence | 1.0630 | 1.85 | 0.0680 |  |
| **Model Fit** | **F (5, 76) = 3.2769; p < 0.0102; R^2^ = 0.1875** | | |  |
| **Variable** | **b** | **t** | **p** |  |
| Intercept | 0.9866 | 0.88 | 0.3824 |  |
| HCV status | 0.1583 | 0.28 | 0.7800 |  |
| Any Substance Dependence | 1.0187 | 1.86 | 0.0663 |  |
| IL5 | 0.0593 | 2.09 | 0.0398 |  |
| MIP1beta | -0.0114 | -2.33 | 0.0225 |  |
| SCF | 0.0137 | 2.03 | 0.0459 |  |
| **G. Pain Interference (BPI-PI)** | | | |  |
| **Model Fit** | **F (2, 75) = 3.9404; p < 0.0237; R^2^ = 0.0974** | | |  |
| **Variable** | **b** | **t** | **p** |  |
| Intercept | 0.3046 | 0.42 | 0.6733 |  |
| HCV Status | 0.5731 | 1.15 | 0.2556 |  |
| Any Substance Dependence | 0.9507 | 1.89 | 0.0626 |  |
| **Model Fit** | **F =(4, 75) = 3.8192; p < 0.0072; R^2^ = 0.1771** | | |  |
| **Variable** | **b** | **t** | **p** |  |
| Intercept | 0.6200 | 0.73 | 0.4691 |  |
| HCV status | 0.7499 | 1.53 | 0.1309 |  |
| Any Substance Dependence | 0.9262 | 1.90 | 0.0615 |  |
| CRP | 0.2556 | 1.93 | 0.0581 |  |
| MMP3 | -0.2944 | -2.09 | 0.0406 |  |
| ^a^Regression models were developed in order to find which combination of plasma immune factors were significantly predictive of neuropsychiatric symptom severity on each of the seven neuropsychiatric variables within the total sample. Models were constructed with a backward selection linear regression of 33 immune factors. The backward selection started with the 33 immune factors and systematically eliminated from the model variables that were not significant, retaining only those with p values (p < 0.10). Based on the final solution of the backward regression, a two step model for each neuropsychiatric variable (DV) was constructed and are presented above; fit parameters are presented as well as the unstandardized regression weights (b), t values and p values for each immune factor. In these models, the first step consisted of regressing the DV onto HCV status (coded 0 for the HCV- control HCV Status, and 1 for the HCV+ HCV Status) and Any Substance (alcohol or other drug) Dependence (from the MINI in which a positive dependence was coded as a 1, otherwise coded as zero). In the second step, the significant immune factors from the backward selection were entered simultaneously with HCV status and Any Substance Dependence (both of which were locked and could not be eliminated) to create the final model. See **Table 1** for immune factor abbreviations. BDI-II = Beck Depression Inventory-II. BPI-PI = Brief Pain Inventory-Pain Interference. BPI-PS = Brief Pain Inventory-Pain Severity. DV = Dependent Variable. FSS = Fatigue Severity Scale. GADI = Generalized Anxiety Disorder Inventory. HCV+ = Adults with chronic hepatitis C virus infection. HCV- = Adults with no history of infection with the hepatitis C virus. | | | | |
